# A Challenge for a Male Noctuid Moth? Discerning the Female Sex Pheromone against the Background of Plant Volatiles

**DOI:** 10.3389/fphys.2016.00143

**Published:** 2016-04-25

**Authors:** Elisa Badeke, Alexander Haverkamp, Bill S. Hansson, Silke Sachse

**Affiliations:** Department of Evolutionary Neuroethology, Max Planck Institute for Chemical EcologyJena, Germany

**Keywords:** *Heliothis virescens*, pheromone-guided flight behavior, plant volatiles, wind tunnel, GC-MS

## Abstract

Finding a partner is an essential task for members of all species. Like many insects, females of the noctuid moth *Heliothis virescens* release chemical cues consisting of a species-specific pheromone blend to attract conspecific males. While tracking these blends, male moths are also continuously confronted with a wide range of other odor molecules, many of which are plant volatiles. Therefore, we analyzed how background plant odors influence the degree of male moth attraction to pheromones. In order to mimic a natural situation, we tracked pheromone-guided behavior when males were presented with the headspaces of each of two host plants in addition to the female pheromone blend. Since volatile emissions are also dependent on the physiological state of the plant, we compared pheromone attraction in the background of both damaged and intact plants. Surprisingly, our results show that a natural odor bouquet does not influence flight behavior at all, although previous studies had shown a suppressive effect at the sensory level. We also chose different concentrations of single plant-emitted volatiles, which have previously been shown to be neurophysiologically relevant, and compared their influence on pheromone attraction. We observed that pheromone attraction in male moths was significantly impaired in a concentration-dependent manner when single plant volatiles were added. Finally, we quantified the amounts of volatile emission in our experiments using gas chromatography. Notably, when the natural emissions of host plants were compared with those of the tested single plant compounds, we found that host plants do not release volatiles at concentrations that impact pheromone-guided flight behavior of the moth. Hence, our results lead to the conclusion that pheromone-plant interactions in *Heliothis virescens* might be an effect of stimulation with supra-natural plant odor concentrations, whereas under more natural conditions the olfactory system of the male moth appears to be well adapted to follow the female pheromone plume without interference from plant-emitted odors.

## Introduction

Odors present in the environment provide information that is crucial for insect survival and reproduction. Most insects use these olfactory cues for finding food, identifying suitable oviposition sites and communicating with their mates. Volatiles that are emitted by plants represent major cues with which an insect detects suitable host plants (Visser, 1986; Bruce et al., 2005), while pheromones are used for intraspecific identification and communication. Lepidoptera males, for example, are able to detect conspecific females releasing a species-specific pheromone blend. In the heliothine moth *Heliothis virescens* (Lepidoptera, Noctuidae), it has been shown that females produce a complex blend of up to seven components in their pheromone glands (Roelofs et al., 1974; Tumlinson et al., 1975; Klun et al., 1979; Pope et al., 1982). Wind tunnel and field experiments have shown that the behavioral activity of this pheromone blend depends highly on the ratio of its individual components (Vetter and Baker, 1983; Ramaswamy and Roush, 1986; Vickers et al., 1991). The pheromone blend is detected by specialized olfactory sensory neurons (OSNs) housed in sensilla trichoidea on the male antenna (Almaas and Mustaparta, 1990, 1991; Berg et al., 1995; Vickers et al., 2001). These OSNs send their axons to the antennal lobe (AL), which represents the primary olfactory processing neuropil, consisting of an array of olfactory glomeruli. Sex pheromone information is processed in a male-specific part of the AL (Hansson and Anton, 2000), the macroglomerular complex (MGC), which in male *Heliothis virescens* comprises four glomeruli (Christensen and Hildebrand, 1987; Hansson et al., 1992, 1995; Vickers and Baker, 1996; Berg et al., 1998; Vickers et al., 1998). The remaining, so-called ordinary, glomeruli process the information of all other odorants including plant and fruit volatiles (Galizia et al., 2000; Hillier and Vickers, 2007). This segregation of the olfactory pathway is partially maintained in the higher brain centers, such as the lateral horn (Zhao et al., 2014).

*Heliothis virescens* is a pest species, and feeds on many plants and crops such as cotton, tomato, soybean, tobacco and chickpea (Fitt, 1989; Cunningham and Zalucki, 2014). Several studies have shown that the olfactory system of both males and females is able to detect and process many volatiles emitted by these host plants (Loughrin et al., 1990; Tingle and Mitchell, 1992; Stranden et al., 2003; Rostelien et al., 2005; Hillier et al., 2006; Hillier and Vickers, 2007). Notably, the chemical diversity of volatile compounds found in all the floral scents investigated so far has been estimated to more than 1700 chemicals (Knudsen et al., 2006). Furthermore, the volatile composition of plants can change depending on environment and stress (reviewed by Dicke and Van Loon, 2000; Beyaert and Hilker, 2014). Damaged plants often emit different volatiles as well as different ratios of the volatile composition compared to undamaged plants. Considering this enormous diversity of chemical compounds, finding a sexual partner in such a complex environment is a big challenge for male moths. They have to detect minute amounts of the conspecific female pheromone blend against a constant background of many other odors. Although pheromone compounds are processed in a separate part of the olfactory system, it has been shown in several moth species that plant volatiles can influence pheromone detection and *vice versa* (Chaffiol et al., 2014; Deisig et al., 2014). Interestingly, plant compounds can even enhance the detection of pheromone components. For example, in the corn earworm *Helicoverpa zea*, simultaneous application of plant odorants with the major sex pheromone component of the moth increases the firing rate of pheromone-responsive OSNs in males, although those neurons do not respond to stimulation with plant odorants separately (Ochieng et al., 2002). Moreover, in beetles (Nakamuta et al., 1997) and many lepidopteran species (Dickens et al., 1993; Light et al., 1993; Reddy and Guerrero, 2000; Deng et al., 2004; Namiki et al., 2008; Schmidt-Büsser et al., 2009; Gurba and Guerin, 2015) the behavioral response is also increased when plant compounds are combined with the corresponding pheromone components. In contrast, a variety of studies demonstrated that pheromone detection can also be inhibited by interactions with plant odorants (Den Otter et al., 1978; Kaissling and Bestmann, 1989; Pophof and Van Der Goes Van Naters, 2002; Party et al., 2009, 2013; Hillier and Vickers, 2011; Chaffiol et al., 2012; Deisig et al., 2012; Pregitzer et al., 2012; Hatano et al., 2015). Hatano et al. (2015) showed this inhibitory effect even at the behavioral level. These contradictory findings give raise to the question whether the olfactory background is modulating the intraspecific communication of insects. Indeed, in *Heliothis virescens*, certain plant-emitted volatiles reduce the detection of *Z*11-16:Ald, the major sex pheromone component, at the level of the pheromone receptor HR13 (Pregitzer et al., 2012). Single sensillum recordings of *Z*11-16:Ald-tuned OSNs concur with this inhibitory effect (Hillier and Vickers, 2011). Moreover, in the same study, a suppressive effect for OSNs being tuned to the minor component *Z*9-14:Ald could be demonstrated. However, whether these effects at the sensory level are maintained throughout the olfactory system and thus may affect male moth behavior is unknown. We therefore analyzed whether a background of plant volatiles influences pheromone-guided behavior in *Heliothis virescens* using wind tunnel experiments. We analyzed the impact of complete and naturally occurring odor blends as well as of individual plant volatiles at different concentrations. Furthermore, we quantified the volatile emissions of all stimuli using gas chromatography analysis. Surprisingly, we observed pheromone-plant interactions only at high and supra-natural odor concentrations. We therefore conclude that pheromone-plant interactions in *Heliothis virescens* might not occur under natural conditions and that male moths are able to detect their conspecific female against a complex background of plant volatiles.

## Materials and methods

### Insect rearing

We obtained *Heliothis virescens* from the Department of Entomology in the Max Planck Institute of Chemical Ecology in Jena. Moths originated from Clemson University in Clemson, South Carolina. These were maintained at the institute for several generations, where they were reared as follows: Eggs of *H. virescens* were gained from single pair matings in 0.5 l cups. In order to minimize inbreeding depression, females and males of different families were chosen. A mesh on top of the mating cups allowed the females to oviposit their eggs. Larvae were subsequently maintained in 10-cm Petri dishes containing artificial pinto bean diet (Burton, 1979). They were separated at second instar. After eclosion, about 15–20 males of the same age were segregated into 30 × 30 × 30 cm rearing cages. A 10% sucrose solution was provided *ad libitum*. Animals were kept at 60% rel. humidity and at 23–25°C under a 16:8 h light-dark cycle. The light level during scotophase was 0.4 lux. 2- to 6-day-old virgin male moths were used for behavioral experiments.

### Plant material

In order to use the headspaces of whole plants for volatile collection and behavioral experiments, cotton (*Gossypium hirsutum*) and tomato plants (*Solanum lycopersicum*) were grown individually in 1-liter pots in the greenhouse at 23–25°C and 50–70% rel. humidity under a 16:8 h light-dark cycle. After the beginning of their elongation stage and until the experiments were performed, plants were transferred to a climate chamber providing 22–25°C and 60–70% relative humidity. They were watered daily with 100 ml tap water supplemented with 0.12 g^*^ml-1 fertilizer. For the experimental approach undamaged or damaged plants were taken. In order to damage plants, four to five third- and fourth-instar larvae of *H. virescens* were allowed to feed on the plant before the behavioral assay was conducted. Larvae were removed from the plants after 24 h.

### Behavioral approach

#### Wind tunnel

**Figure 1:**
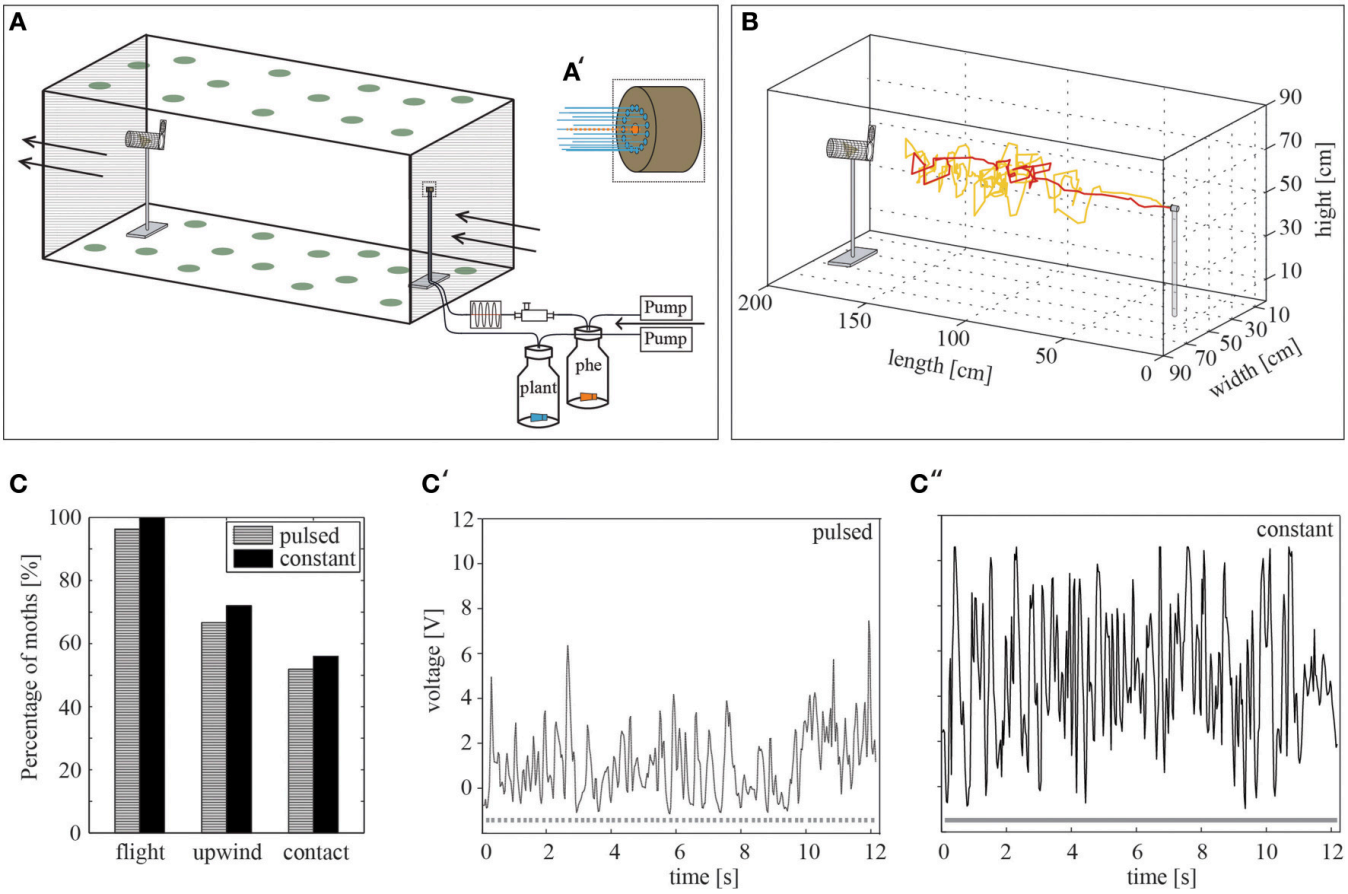
**The wind tunnel system**. **(A)** Schematic representation of the wind tunnel system including the stimulus device. The ceiling and the floor were covered by green dots in order to provide a pattern for the insects to orient on. Arrows indicate the air stream. An air flow is transported via pumps through the stimulus bottles and released by the stimulus outlet. The pheromone-loaded air is pulsed beforehand at 10 Hz by using a cross-valve. phe = pheromone **(A')** Magnification of the stimulus outlet (dashed square). The dotted orange line represents the middle nozzle, which emits a pulsed pheromone stimulus, while the blue lines highlight the constant plant odor flow released by the surrounding nozzles. **(B)** Two representative flights of different males (yellow, red) toward the pheromone blend. **(C)** The percentage of male *H. virescens* attempting flight behavior, achieving upwind flight and making source contact is similar for constant (*N* = 25) and pulsed (*N* = 27) pheromone stimulation (*p* > 0.05, Fisher's exact test). **(C',C**″**)** Visualization of the constant and pulsed odor plume using a photoionization detector (PID) at 110 cm distance from the stimulus outlet. Dotted and continuous lines below the curves represent the odor stimulation. Fewer volatiles can be detected in the pulsed **(C**′**)** odor plume than in the constant plume **(C**′**')**. PID measurements: U_pulsed_ = 1.81 V, U_constant_ = 4.77 V.

Insects were tested in a 220 × 90 × 90 cm Plexiglas wind tunnel (Figure 1A) under infrared and red light conditions with a white light supply of 0.4 lux. A purified, humidified and tempered airflow of 0.27 m/s was blown through the wind tunnel, providing 23°C and 60–70% relative humidity.

#### Stimulus device

For synthetic odorants the odor plume was created by connecting separately two 50 ml glass bottles via *Teflon* tubing to the stimulus outlet on a stick 55 cm long (Figure 1). The distance to the upwind end of the wind tunnel was 23 cm. Pumps, which sucked the ambient air through a charcoal filter for cleaning, generated a stimulus flow of 0.48–0.50 l/min through the tubing leaving the bottles. In each of the bottles, a rubber septum loaded with the test odorants was inserted. The bottle, which contained the pheromone blend, was additionally connected to an *Arduino* microprocessor-controlled cross-valve before being released by the middle nozzle (ID 1 mm) of the stimulus outlet (Figure 1A′). Thus, pulsed stimulations of 10 Hz could be achieved. It has been shown that pulsed stimulation affects the flight behavior of male moths in the wind tunnel (Vickers and Baker, 1994). We therefore compared pheromone attraction to either a constant pheromone plume or a pulsed pheromone plume using an optimal pulse frequency of 10 Hz (Figure 1C). The second stimulus bottle was connected to the circular arranged nozzles (ID 0.5 mm each). For experiments using the headspaces of different plants, a glass cylinder (10 l) containing a plant was connected to the system instead of to the second stimulus bottle. A Teflon disc on the bottom with a central opening separated green plant material from soil and roots. Compressed, charcoal-filtered air with a flow of 1 l/min was inserted into the cylinder. Only 0.48–0.54 l/min of the cylinder headspace was sucked via a pump into to the wind tunnel.

#### Animal handling

All experiments were performed 2–7 h during scotophase, when pheromone responsiveness is highest (Shorey and Gaston, 1965). At least 1 h before testing, male moths were transferred individually into Ø 7 × 10 cm mesh tubes and placed in a small room near the wind tunnel that had the same conditions. Active moths were chosen for testing. At the beginning of each experiment, a mesh tube containing a moth was inserted into a releasing device in the odor plume at the downwind end of the wind tunnel. The releasing device was controlled via the microprocessor in order to open the cage automatically 2 min after placing the moth in the mesh tube. Flight behavior was subsequently recorded for 5 min. After the first source contact within this time interval, males' behavior was tracked for 2 min.

#### 3-D video tracking

During the experiment the releasing device, all wind tunnel conditions and the flight paths were computer-controlled from a separate room. In order to observe odor-guided flight behavior, we used a custom-built video tracking system. Four cameras (C615, Logitech, Newark, NJ, USA, 800 × 600 pixels, 0.3 cm^2^ pixel size), which were located at the side and on the top of the wind tunnel, recorded the flight path of each moth. By using a background subtraction algorithm, the position of each moth was calculated at a rate of 10 Hz. A fifth camera, which was attached to the upwind end of the wind tunnel, allowed the recording of males' behavior close to the odor source.

#### Determining optimal conditions for the wind tunnel

In order to monitor pheromone attraction and to study whether it is influenced by background volatiles, we started to find the best conditions for the bioassay. A stimulus device was used to create a point source emitting either a pulsed or a constantly emitted pheromone blend of *Heliothis virescens* together with a surrounding odor plume of a constant solvent release (Figure 1A′). When stimulating with the conspecific pheromone blend, male moths showed clear pheromone-guided upwind flight behavior. This behavior can be characterized by locking on to the pheromone plume followed by upwind flight, zigzagging, casting behavior and, finally, contact with the source (Figure 1B). When placed in a constant or a pulsed pheromone plume, all moths started their flight within 5 min. (Figure 1C). Hence, the type of stimulation influenced neither the percentage of moths attempting upwind flight nor the number of source contacts. In order to compare the pulsed and constant odor plume structure, we measured the presence of volatiles using a photoionization detector (PID). The results showed that the probability that a moth hits a volatile in a pulsed odor plume is less than the probability that a moth hits one in a constant plume (Figures 1C′,C″). However, although the odor plume structure was different, pheromone attraction was similar for both odor applications. We chose pulsed pheromone stimulation for all subsequent experiments in our study.

### Odorants

All synthetic odorants tested were commercially available and acquired from Sigma (http://www.sigma-aldrich.com), Bedoukian (http://www.bedoukian.com) or pherobank (http://www.pherobank.com). They were obtained in the highest available purity. β-caryophyllene (CAS 87-44-5, purity > 98.5%), racemic linalool (CAS 78-70-6, purity > 97%) and (*Z*)3-hexen-1-ol (CAS 928-96-1, purity > 98%) are well-described plant compounds. They are detectable by male and female *Heliothis virescens* (Paré, 1997; De Moraes et al., 2001; Skiri et al., 2004; Rostelien et al., 2005; Hillier and Vickers, 2007), and they have been used previously in studies investigating plant-pheromone interaction on *H. virescens* (Dickens et al., 1993; Hillier and Vickers, 2011; Pregitzer et al., 2012).

A synthetic pheromone blend, which contained the seven components, (*Z*)-11-hexadecenal (*Z*11-16:Ald, CAS 53939-28-9, purity 97-98%), (*Z*)-9-tetradecenal (*Z*9-14:Ald, CAS 53939-27-8, purity > 93%), tetradecenal (14:Ald, purity > 98%), hexadecanal (16:Ald, CAS 629-80-1, purity > 93%), (*Z*)-7-hexadecenal (*Z*7-16:Ald, CAS 56797-40-1, > 95%), (*Z*)-9-hexadecenal (*Z*9-16:Ald, CAS 56219-04-6, purity > 90%) and (*Z*)-11-hexadecenol (*Z*11-16:OH, CAS 56683-54-6, purity > 98%), was used (Roelofs et al., 1974; Tumlinson et al., 1975; Klun et al., 1979). We prepared the blend relative to *Z*11-16:Ald (100%) and added 5% *Z*9-14:Ald, 5% 14:Ald, 10% 16:Ald, 2% *Z*7-16:Ald, 2% *Z*9-16:Ald and 1% *Z*11-16:OH of the compounds (Pope et al., 1982), in order to test the sexual attraction of *H. virescens* males toward their conspecific pheromone blend. Tetradecenal was synthesized from commercially available tetradecanol (Sigma) by the Research Group Mass Spectrometry/Proteomics in the Max Planck Institute of Chemical Ecology in Jena.

Both synthetic plant compounds and the pheromone blend consisted additionally of 1.25% of the antioxidant 3.5-Di-tert-butyl-4-hydroxytoluene (BHT, CAS 128-37-0, purity ≥ 99%, Sigma). They were subsequently pipetted on individual rubber septa (Thomas Scientific, http://www.thomassci.com/). Before being used, rubber septa were cleaned with hexane (CAS 110-54-3, Sigma), which was furthermore used as a solvent for all odorants. For plant components, concentrations between 30 and 300 μg/μl were used. The pheromone blend was adjusted to *Z*11-16:Ald with a concentration of 300 μg/μl. We always indicate the final concentration for each rubber septum.

### Volatile collection, analysis, and quantification

In order to quantify the actual amount of volatiles being released by the rubber septum and pumped through the tubing into the wind tunnel, we used polydimethylsiloxane (PDMS) tubes (OD 2.3 mm, Reichelt Chemietechnik, http://www.rct-online.de). By introducing the PDMS tubes for 2 h into the odor flow close to the stimulus outlet, we could collect volatiles during testing. Volatiles being released by plants were collected with the same approach. Samples were stored at -20°C until use. All samples were examined on an Agilent 7890A gas chromatograph (Agilent Technologies, CA) running in splitless mode and being connected to an Agilent 5975C mass spectrometer (electron impact mode, 70 eV, ion source: 230°C, quadrupole: 150°C, mass scan range: 33–350 u). We used a nonpolar column (HP-5 MS UI, 30 m length, 0.25 mm ID, 0.25 μm film thickness, J and W Scientific) under constant helium flow of 1.1 ml/min. The GC oven was programmed to hold 40°C for 3 min, to increase the temperature at 5C°/min to 200°C, then to increase temperature at 20°C/min to 260°C. The maximum temperature was held for 10 min. For identification, mass spectra were compared with Kovats retention time indices to reference compounds or to those published by the National Institute of Standards and Technologies (NIST, version 2.0). Retention times for all compounds were determined by using standards. Quantifications of emission rates were subsequently calculated based on the comparison of the internal standard of 10 ng/μl 1-Bromohexane (CAS 111-25-1, purity 98 %, Sigma) and peak area of single compounds.

### Data analysis and statistics

Microsoft Excel, Gnu R, custom-written Matlab scripts (MATLAB version- Mathworks, USA) and Adobe Illustrator were used in order to analyze and plot all data. Statistics were performed with the software Gnu R and GraphPad Instat. We calculated the emission rate of volatiles being released within 1 h for each compound based on the internal standard by using the commercial software GC ChemStation (Agilent Technologies) and Microsoft Excel.

In order to investigate the attractiveness of volatiles in the wind tunnel, we calculated the percentage of moths (1) starting to fly, (2) achieving upwind flight, and (3) contacting the source for each group of odor stimulation. An odor plume was called attractive if moths reached and contacted the odor source. In order to investigate pheromone-plant interaction, we further examined the average number of source contacts per male out of all individual moths within a group for the test period. We quantified the number of contacts for another 2 min after the first contact. Males without contacts were counted as zeros. For statistical analysis, the group tested with the pheromone blend alone was always taken as a control group. The percentage of moth within a test group was compared to the pheromone group by means of Fisher's exact test, with a Bonferroni-Holm correction. The number of source contacts was evaluated using the Kruskal-Wallis test with Dunn's multiple comparisons test. The pheromone-guided flight behavior of each attracted male was analyzed in more detail by calculating the percentage of relative abundance of flight angles in y- and z-direction and the average upwind speed within an 80 cm distance from the stimulus outlet. Both angles and upwind speed were measured with an interval of 10 Hz. The last 10 cm of the track were excluded due to the fact that it could not be tracked reliably in all moths. Animals which performed zigzagging and casting movements possessed flight angles greater than zero degrees. Angles around zero degrees exhibit straight upwind movement. Upwind speed (cm/s) is the speed of an animal relative to the odor source. Positive values indicate upwind movement, negative values downwind movement, while values around zero indicate cross-wind movement. The Kruskal-Wallis test and Dunn's multiple comparisons test were used for statistics.

## Results

### Host plant headspaces did not affect pheromone attraction

**Figure 2:**
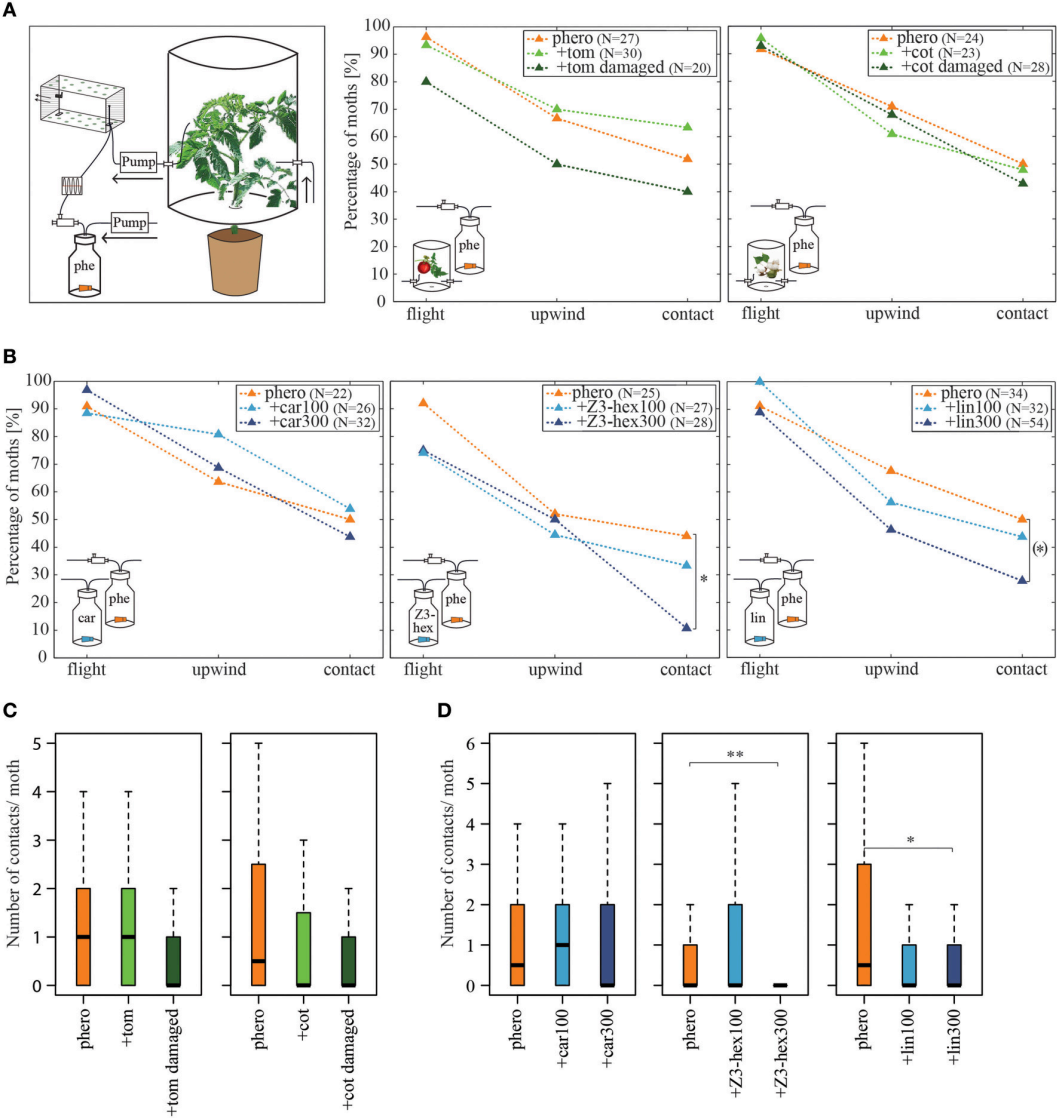
**Influence of host plant headspaces on pheromone-guided flight behavior**. **(A)** Percentage of moths attempting flight behavior, achieving upwind flight and making source contact, when simultaneously stimulated with the pheromone blend and a tomato (middle panel) or cotton (right panel) plant headspace. Plants were intact or damaged by larvae. The left panel highlights the changes in the odor stimulation device. The headspace of the plants was sucked via a pump through the wind tunnel. The pulsed pheromone stimulation was implemented as described in Figure 1. There was no significant difference in pheromone attraction when insects were stimulated simultaneously with undamaged or damaged tomato or cotton headspaces compared to pheromone stimulation alone (*p* > 0.05, Fisher's exact test, Bonferroni-Holm correction). **(B)** Percentage of moths attempting flight behavior, achieving upwind flight and making source contact, when simultaneously stimulated with the pheromone blend and the synthetic odorants β-caryophyllene (left panel), (*Z*)3-hexenol (middle panel) or linalool (right panel) each in two different concentrations (100 and 300 μg/μl). While β-caryophyllene did not affect pheromone-guided flight behavior, high concentrations of (*Z*)3-hexenol decreased the amount of moths contacting the source. A similar tendency was observed for linalool. Asterisks represent significant differences (*p* < 0.05, Fisher's exact test with Bonferroni-Holm correction). The bracket indicates significant differences without Bonferroni-Holm correction (*p* = 0.0426). **(C)** Number of contacts per individual moth for all tested males from **(A)**. No differences in the number of contacts when different plant headspaces were used (*p* > 0.05, the Kruskal-Wallis test, Dunn's multiple comparisons test). **(D)** Number of contacts per individual moth for all tested males from **(B)**. Moths had significantly fewer contacts when high dosages of (*Z*)3-hexenol or linalool were applied to the septa than when they were not (*p* < 0.05, the Kruskal-Wallis test, Dunn's multiple comparisons test). car, β-caryophyllene; cot, cotton; lin, linalool; phe/phero, pheromone; tom, tomato; *Z*3-hex, (*Z*)3-hexenol.

Since it has been shown that different plant-emitted volatiles affect detection of the major sex pheromone component *Z*11-16:Ald in male *Heliothis virescens* at the physiological level (Hillier and Vickers, 2011; Pregitzer et al., 2012), we tested whether behavioral performance is similarly affected. In order to provide a naturally occurring odor source, we used the headspaces of two host plants, tomato and cotton, to examine their influence on pheromone-guided flight behavior (Figure 2A, left panel). First, we tested the headspaces of the two host plants alone. We observed that both the tomato headspace as well as the cotton headspace induced only very low degrees of upwind flight and source contact (*N* = 17–20, upwind 1–3 moths, contact 0–1 moth; data not shown). We next applied the conspecific pheromone blend to each plant headspace simultaneously. The results reveal that a pheromone plume with a background of either tomato (Figure 2A, middle panel) or cotton headspace (Figure 2A, right panel) showed similar attractiveness as compared to a pheromone blend with no plant odor background. The number of source contacts was also not affected (Figure 2C, Table 1). Hence the pheromone-guided flight was not influenced by the presence of a naturally occurring plant odor blend.

**Table 1:** **Effect of intact and damaged tomato and cotton plants on pheromone-guided flight behavior**.

**Stim. 1**	**Stim. 2**	**Sample size**	**Flight [%]**	**Upwind [%]**	**Source contact [%]**	**Upwind speed [cm/s] ± SD**	**Number of contacts ± SD**
–	Phero	27	96.3	66.7	51.9	25.8 ± 29.6	1.19 ± 1.71
Tom	Phero	30	93.3	70	63.3	22.7 ± 27.3	1.37 ± 1.56
Tom damaged	Phero	20	80	50	40	24 ± 22.7	0.6 ± 0.99
–	Phero	24	91.7	70.8	50	24 ± 22.7	1.75 ± 2.67
Cot	Phero	23	95.7	60.9	47.8	30.5 ± 23	1 ± 1.38
Cot damaged	Phero	28	92.9	67.9	42.9	25.1 ± 33.6	0.75 ± 1.17

*Number of tested individuals and the percentages of male moths, for the experiments shown in Figures 2A,C, which started their flight, showed upwind movement and had source contact; also their upwind speed. The last column represents the number of contacts for all tested males. Stimulus (stim.) 1 and 2 together form the odor plume. Odorants of stimulus 1 were emitted continuously, while stimulus 2 (pheromone) was pulsed. A (−) in stimulus 1 represents the use of a solvent instead of an odorant. SD, standard deviation*.

*no significant differences within a column to the solvent-pheromone stimulation (p > 0.05, Fisher's exact test with Bonferroni-Holm correction; Number of contacts and upwind speed: Kruskal-Wallis with Dunn's multiple comparisons test)*.

*cot, cotton; phero, pheromone; tom, tomato*.

It has been shown that larval damage influences the composition and/or the emission rate of plant volatiles (De Moraes et al., 1998). The attraction of female moths to a damaged plant headspace depends on the amount of herbivore-induced plant volatiles (Späthe et al., 2013). In order to examine whether herbivore damage significantly influences pheromone detection, we let four to five larvae feed on both host plants and tested the attractiveness of the induced headspace in our wind tunnel. Only three moths at most moved upwind when placed in a damaged tomato or cotton odor plume, but none of them contacted the source (*N* = 15–17; data not shown). When a damaged tomato plant headspace was presented together with the pheromone blend, we observed that 12% fewer individuals reached the source as compared to the pure pheromone blend (Figure 2A, middle panel, Table 1). However, this decrease was not significantly different from the response to the pheromone blend without background. Likewise, moths flying in a pheromone plume did not contact the source significantly more often (Figure 2C, Table 1). The same applies for the cotton headspace: larval damage in cotton plants affected neither pheromone-guided flight behavior nor the number of odor source contacts (Figure 2A, right panel, Figure 2C, Table 1).

**Figure 3:**
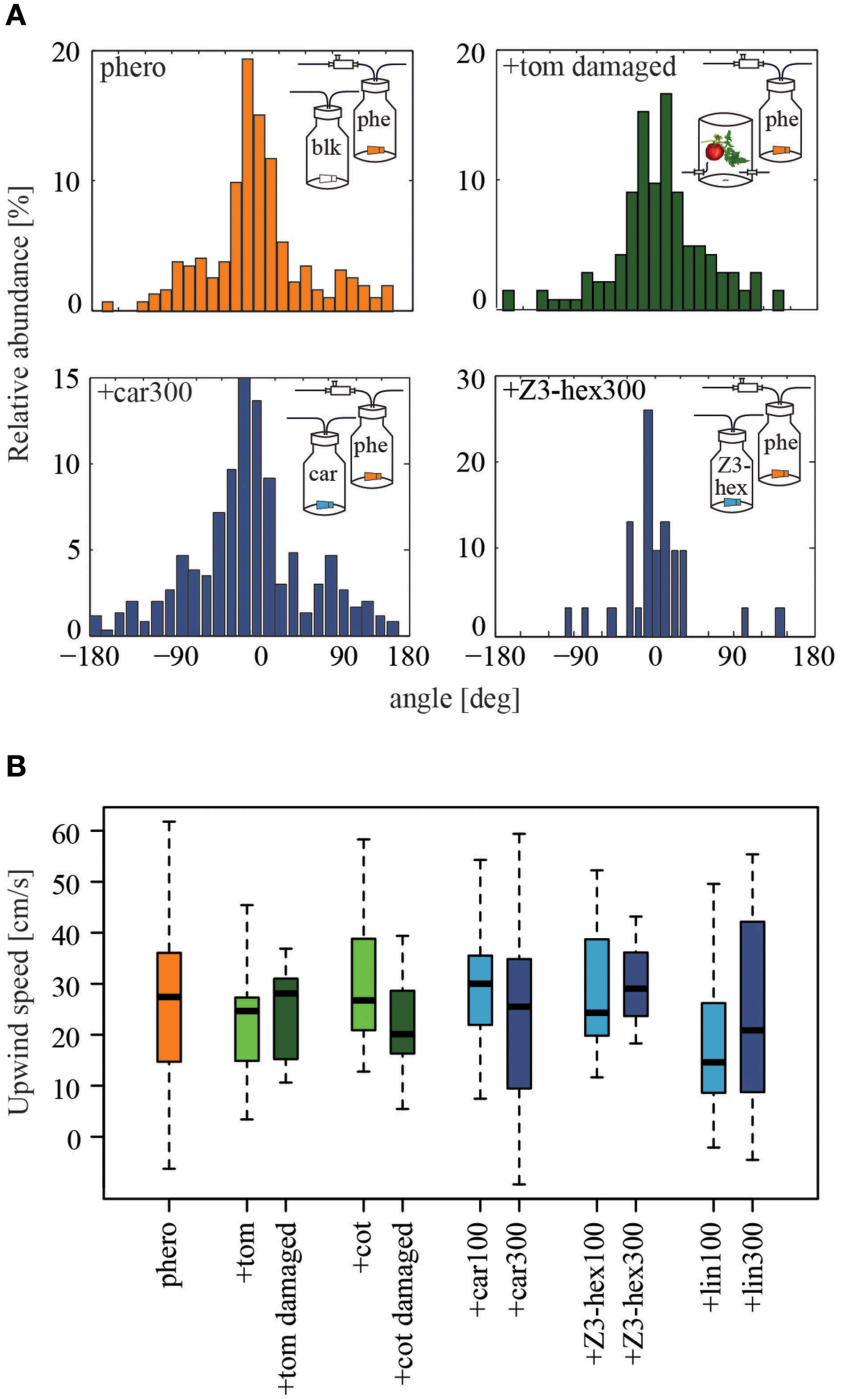
**Influence of individual plant volatiles on pheromone-guided flight behavior**. **(A)** Relative abundance of flight angles being performed by male moths within the upwind section of the wind tunnel close to the source, when pheromones alone (orange), a pheromone and the headspace of a damaged tomato plant (dark green), pheromone and β-caryophyllene or (*Z*)3-hexenol (dark blue, both with 300 μg), were used for stimulation. The distribution histograms cumulate all tracks of moths contacting the stimulus outlet. Flight angles are shown as cumulated azimuth and zenith angles. Most of the time insects facing upwind in the odor plume they perceived performed zigzagging and casting activities. **(B)** Males moths showed similar upwind speeds (*p* > 0.05, the Kruskal-Wallis test, Dunn's multiple comparisons test), when stimulated with the pheromone alone (orange, *N* = 41), or the pheromone in combination with 100 μg (N_car_= 12, N_Z__3−hex_ = 8, N_lin_ = 9) or 300 μg (N_car_ = 13, N_Z__3−hex_ = 3, N_lin_ = 14) of artificial odorants (blue), or with the headspaces of cotton (N_intact_ = 8, N_dam_ = 13), or tomato plants (N_intact_ = 10, N_dam_ = 7) (green). blk, blank; car, β-caryophyllene; cot, cotton; lin, linalool; phe/phero, pheromone; tom, tomato; *Z*3-hex, (*Z*)3-hexenol.

In order to analyze pheromone-guided flight behavior in more detail, we dissected the flight mechanism. We asked how males manoeuver in response to an odor source and if their flight patterns are influenced by different odor plumes. We therefore examined the flight angles of attracted individuals as well as individual's upwind speed (Figure 3). In Figure 3A the relative abundance of flight angles for male moths in a pure pheromone plume and a tomato-pheromone plume are representative examples. Independent of odor stimulation, the most abundant flight angles of male *Heliothis virescens* were around zero degrees, indicating a relatively straight upwind flight. Angles up to ±180° represented additional zigzagging and casting behavior. Analysis of the upwind speed of the attracted insects resulted in values around 27 cm/s regardless of the odors present in the plume (Figure 3B, Table 1). In summary, we observed that neither the number of source contacts nor the flight pattern was affected when a complete plant headspace was applied simultaneously with the pheromone blend.

### Certain plant-emitted volatiles reduced pheromone attraction

Interestingly, we did not observe the significant reduction in pheromone-elicited flight behavior suggested in previous studies. These however reported plant-pheromone interactions in moths using single plant-related compounds instead of complete headspaces. In order to analyze whether single plant volatiles could affect the pheromone response, we tested the three plant-emitted volatiles, β-caryophyllene, (*Z*)3-hexenol and linalool, each in two different concentrations based on the study by Pregitzer et al. (2012). As a side note, all of these compounds are up-regulated in larval-damaged plants (Paré, 1997; De Moraes et al., 1998, 2001; Stranden et al., 2003; Morawo and Fadamiro, 2014).

In comparison to pure pheromone stimulation, both concentrations of β-caryophyllene in combination with the pheromone did not reduce the attractiveness of the pheromone (Figures 2B,D, left panels, Table 2); moreover, β-caryophyllene alone did not attract any male moths, independent of its concentration (tested concentrations: 60, 100, 200, 300 μg/μl; *N* = 16–19; data not shown). Likewise, male moths did not respond to (*Z*)3-hexenol alone (100, 300 μg/μl; *N* = 16; data not shown). However, adding 300 μg/μl of (*Z*)3-hexenol to the pheromone plume significantly reduced the number of individuals (by 33%) and their frequency contacting the source, although equal percentages displayed upwind flight (Figures 2B,D, middle panels, Table 2). Interestingly, lowering the concentration of (*Z*)3-hexenol (i.e., 100 μg/μl) did not significantly decrease the moths' response to pheromones. We observed a similar dose-dependent effect when insects were stimulated simultaneously with the pheromone blend and the odor linalool. Linalool alone at concentrations of 30, 60, 100, 200, or 300 μg did not attract males at all and resulted in no upwind flights (*N* = 15–30; data not shown). However, adding the highest concentration of linalool to the pheromone plume resulted in 22% fewer individuals contacting the source compared to the number contacting the source when only the pheromone was used (Figures 2B,D, right panels, Table 2). This effect was also concentration-dependent, since we did not observe any reduction in pheromone-guided flight behavior when we reduced the concentration of linalool.

**Table 2:** **Effect of β-caryophyllene, (*Z*)3-hexenol and linalool on pheromone-guided flight behavior**.

**Stim. 1**	**Stim. 2**	**Sample size**	**Flight [%]**	**Upwind [%]**	**Source contact [%]**	**Upwind speed [cm/s] ± SD**	**Number of contacts ± SD**
–	Phero	22	90.1	63.6	50	22.6 ± 25.5	1 ± 1.23
car100	Phero	26	88.5	80.8	53.8	28.9 ± 28	1.04 ± 1.22
car300	Phero	32	96.9	68.8	43.8	24.7 ± 33.4	1.09 ± 1.53
–	Phero	25	92	52	44	29.1 ± 21.8	1.16 ± 1.84
Z3-hex100	Phero	27	74.1	44.4	33.3	31.1 ± 28.2	1 ± 1.71
Z3-hex300	Phero	28	75	50	10.7^*^	30.2 ± 28	0.27 ± 0.93^**^
–	Phero	34	91.2	67.6	50	15.8 ± 30.2	1.32 ± 1.66
lin100	Phero	32	100	56.3	43.8	16.1 ± 40.4	0.94 ± 1.9
lin300	Phero	54	88.9	46.3	27.8 (^*^)	23.7 ± 34.5	0.63 ± 1.51^*^

*Number of tested individuals and the percentages of male moths, for the experiments shown in Figures 2B,D, which started their flight, showed upwind movement, and had source contact; also, their upwind speed. The last column includes the number of contacts for all tested males. The stimuli were applied as described in Table 1. SD, standard deviation*.

^*^Within a column indicate significant differences to the solvent-pheromone stimulation (

* *p < 0.05*,

** *p < 0.01*,

*Fisher's exact test with Bonferroni-Holm correction*,

(*) *p < 0.05*,

*Fisher's exact test, p > 0.025 with Bonferroni-Holm correction; Number of contacts and upwind speed: Kruskal-Wallis with Dunn's multiple comparisons test)*.

*car, β-caryophyllene; lin, linalool; phero, pheromone; Z3-hex, (Z)3-hexenol*.

We observed similar flight angles in a pheromone plume compared to those in a plume consisting of the pheromone blend and β-caryophyllene, (*Z*)3-hexenol or linalool, as shown for β-caryophyllene and (*Z*)3-hexenol (Figure 3A, Table 2). The distribution histograms represent the cumulated azimuth and zenith angles of all male moths contacting the stimulus outlet. Since we measured less animals for (*Z*)3-hexenol, the histogram shows less cumulated angles. However, the distribution of the angles is similar to those of the other stimuli. Most angles were around zero degrees. Furthermore, males moved upwind to the source with on average 25 cm/s (Figure 3B). In summary, adding certain plant-related compounds at high concentration to the pheromone plume reduced the pheromone-guided response in male *Heliothis virescens* but did not lead to a different flight pattern: neither the flight direction in order to approach the odor source nor the upwind speed was influenced by plant volatiles.

### Concentration quantification of synthetic odorants vs. plant-released volatiles

**Figure 4:**
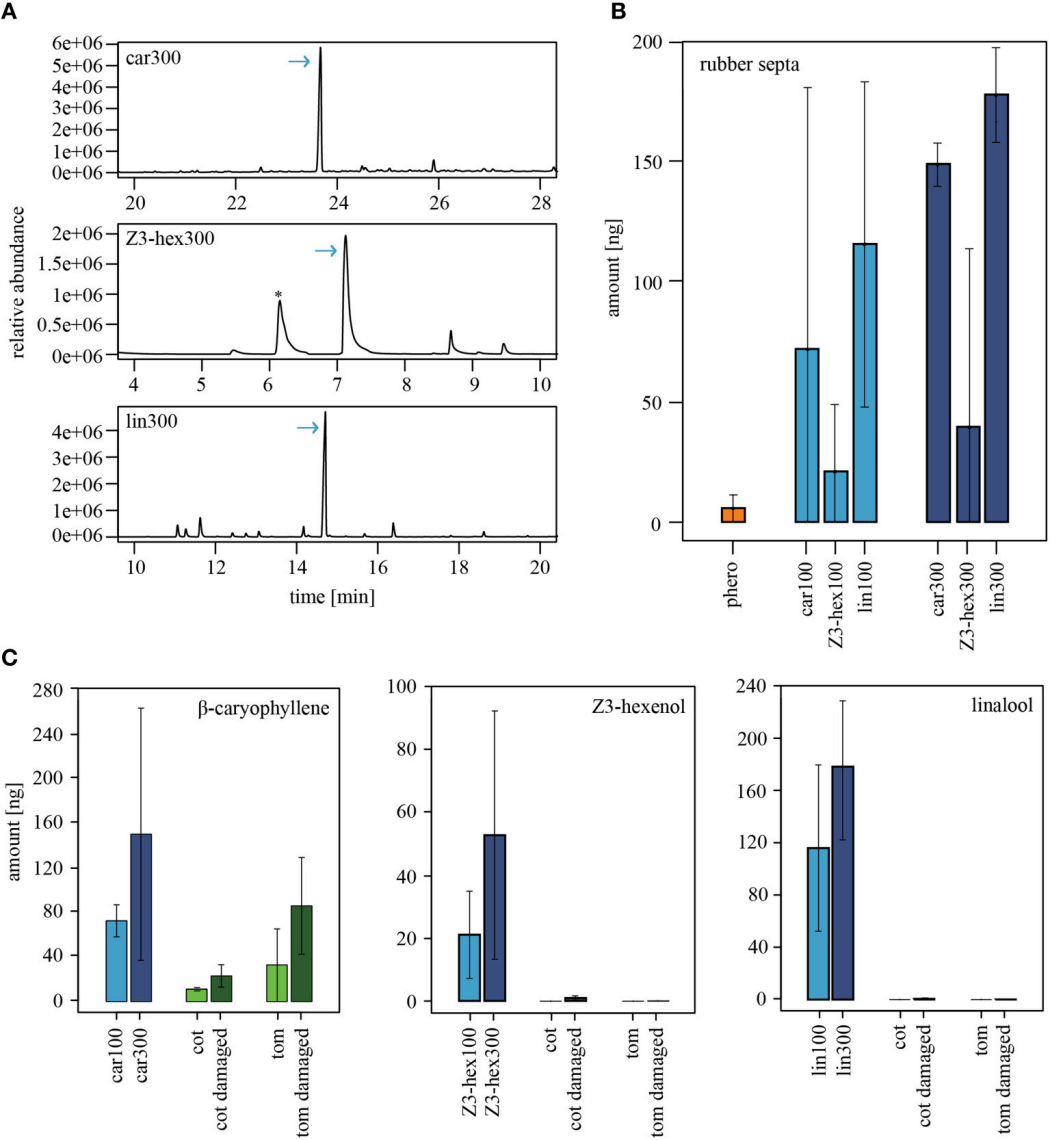
**Emission rates of volatiles from rubber septa and entire plants. (A)** GC-MS example traces showing the relative abundance of the synthetic odorants β-caryophyllene (upper panel), (*Z*)3-hexenol (middle panel) or linalool (lower panel). 300 μg/μl of the odorant were loaded on a rubber septum, and the headspace was collected for 2 h with PDMS tubes. Arrows indicate the corresponding odor peak in the headspace. The asterisk represents the peak of a siloxane, which is a constituents of PDMS tubes. **(B)** Amounts of volatiles, which were released by rubber septa loaded with the pheromone blend (N_300μg_ = 2), β-caryophyllene (N_100μg_ = 3, N_300μg_ = 3), (*Z*)3-hexenol (N_100μg_ = 2, N_300μg_ = 2), or linalool (N_100μg_ = 3, N_300μg_ = 3). The averaged emission rates are shown as bar plots (±SEM). Bars represent odorants used in a concentration of 100 μg/μl (light blue) or 300 μg/μl (dark blue). **(C)** Comparison of the odor amount emitted from the rubber septa shown in **(B)** and the corresponding compounds in the plant headspace of intact (light green) and damaged (dark green) tomato (N_intact_ = 2, N_dam_ = 4) and cotton plants (N_intact_ = 2, N_dam_ = 4). Bars represent the averaged emission rates. Similar amounts of β-caryophyllene (left panel) were found in the odor emitted from the rubber septa and in odors released by the plants. (*Z*)3-hexenol (middle panel) and linalool (right panel) released from the plants were either not detected or occurred in low amounts that were not comparable to the amounts being released by the rubber septa. car, β-caryophyllene; cot, cotton; lin, linalool; phero, pheromone; tom, tomato; *Z*3-hex, (*Z*)3-hexenol.

Our experiments show that only the application of linalool and (*Z*)3-hexenol at high concentration reduced the attractiveness of male *Heliothis virescens* to the pheromone, while the headspace of host plants did not show any influence. In order to analyze whether the difference is just a matter of odor concentration, we quantified the actual amount of the synthetic odorants released by the rubber septa (Figures 4A,B). While 3 ng of the major sex pheromone component *Z*11-16:Ald could be quantified via PDMS tubes, the plant components, β-caryophyllene, (*Z*)3-hexenol and linalool, were measured in much higher amounts. The amount of β-caryophyllene was 3.5-fold higher than the amount of (*Z*)3-hexenol, while the linalool release was 5-fold higher than the amount of (*Z*)3-hexenol. When pipetting three times the concentration on a rubber septum, both plant volatiles resulted in doubled emission rates, while only 1.5-fold of linalool was detected.

Are the synthetic single odor quantities that reduced the attractiveness of pheromones in our wind tunnel studies similar to those released by intact and damaged tomato and cotton plants? To find out, we quantified the release rate of β-caryophyllene, (*Z*)3-hexenol and linalool in damaged and undamaged host plants (Figure 4C). Larval damage in tomato and cotton plants led to an increase of β-caryophyllene (Figure 4C, left panel), and β-caryophyllene was released in quantities comparable to those of the synthetic odorant. However, β-caryophyllene had no effect on pheromone-guided flight behavior in male moths (Figure 2A). In contrast, (*Z*)3-hexenol and linalool could not be detected in undamaged plants or were found in only low quantities in damaged plants (Figure 4C, middle and right panels). This discrepancy shows that the concentrations of (*Z*)3-hexenol and linalool that reduced pheromone attraction (Figure 2A) were much higher than the natural emission of an entire plant. Hence, odorants that influence pheromone-guided behavior in male moths are not emitted in comparable quantities by plants. We therefore conclude that plant-pheromone interactions in *Heliothis virescens* most likely occur only under laboratory conditions, where very high odor concentrations are used.

## Discussion

We show that pheromone-plant odor interactions occur at the behavioral level of male *Heliothis virescens*, similar to those previously observed at the sensory level (Hillier and Vickers, 2011; Pregitzer et al., 2012). However, we also show that these interactions occur only at supra-natural concentrations of certain plant-emitted volatiles. Our findings therefore suggest that, in a natural environment, male moths are able to detect their conspecific female against a complex background of plant volatiles without negative effects on their pheromone-directed flight behavior.

Certain plant-related volatiles interfere with the detection of the major sex pheromone component of *Heliothis virescens* at the pheromone receptor HR13 and thereby reduce the response of pheromone-detecting OSNs in the MGC (Pregitzer et al., 2012). Interestingly, this interference varies for different plant compounds: linalool and (Z)3-hexanol strongly suppress the pheromone response, while other compounds, such as β-caryophyllene, do not lead to any reduction. These findings correlate well with our behavioral results from experiments using the wind tunnel: while β-caryophyllene did not influence pheromone-guided flight behavior, high concentrations of (*Z*)3-hexenol and linalool reduced the attractiveness of the pheromone by at least 22%. Hence our results show that the coding of pheromone-plant interactions at the sensory level corresponds to the altered behavioral responsiveness of male moths. The representation of odor-induced activity in the AL therefore allows a prediction of the behavioral outcome. Notably, a correlation between the representation of odors in the AL and the behavioral performance has already been demonstrated in several species, such as honeybees (Guerrieri et al., 2005), flies (Knaden et al., 2012) and moths (Kuebler et al., 2012).

The behavioral performance of the moth ultimately results from the odor representation in higher brain centers and is determined by the integration of different processing channels within the neuronal network. Interestingly, when the antenna of the male *Heliothis virescens* moth was stimulated with β-caryophyllene and the major sex pheromone component *Z*11-16:Ald, single sensillum recordings showed an enhanced spiking activity compared to the response evoked by *Z*11-16:Ald alone (Hillier and Vickers, 2011). In contrast, when the major pheromone component was exchanged for the minor pheromone component, *Z*9-14:Ald, the pheromone response was suppressed (Hillier and Vickers, 2011). Although β-caryophyllene is influencing the neuronal activity of pheromone-responsive OSNs in the periphery, we did not observe any effect of this plant volatile onto the pheromone-guided flight behavior in our windtunnel experiments. Since β-caryophyllene modulates the major and minor pheromone pathways in opposing directions (Hillier and Vickers, 2011), the detection of the whole pheromone blend, including the two compounds, *Z*11-16:Ald and *Z*9-14:Ald, might not be modulated in the end.

Moreover, in the same physiological study (Hillier and Vickers, 2011), both major and minor sex pheromone components, when blended with the plant volatile linalool or (*Z*)3-hexenol, elicited reduced spiking activity in the corresponding pheromone-responsive OSNs. Likewise, in our wind tunnel assay, when high concentrations of the two plant compounds were added, the attractiveness of the complete pheromone blend was decreased, which resulted in reduced pheromone-guided flight behavior.

The three compounds that we used in our study are not the only volatiles being detected in plant headspaces. It would therefore be interesting to know if and how other plant volatiles, when added to the pheromone blend, influence the pheromone-guided behavior of a moth. This is of particular interest, since it has been observed that some of these green leaf volatiles increase the number of males caught in pheromone traps (Dickens et al., 1993). However, when we tested the whole headspaces of cotton and tomato plants, independently of their physiological condition, we did not find any influence on pheromone-guided flight behavior.

Host plants of *Heliothis virescens* that are damaged by larval feeding release volatiles such as β-caryophyllene, (*Z*)3-hexenol and linalool (e.g., Paré, 1997; De Moraes et al., 1998; Morawo and Fadamiro, 2014). All of these were used in our study. When we quantified the natural emission of these compounds, we realized that, except for β-caryophyllene, these odorants occur in only very low concentrations in the headspace of intact or damaged cotton and tomato plants. Although volatiles are usually emitted in higher amounts during daytime than in the dark (De Moraes et al., 2001), male moths are active in the scotophase. Therefore, they will encounter low concentrations of plant volatiles. When the results from the wind tunnel and GC-MS experiments were combined, we observed that unnaturally high concentrations of (*Z*)3-hexenol and linalool reduced the heliothine moths' attraction to pheromones, while a lower dose, which represents the more natural situation, did not affect the attraction.

Taken together, our study underlines the importance of using natural concentrations in order to investigate the ecological relevance of odorants and their influence on animals' behavior.

## Author contributions

EB and SS together conceived and designed the study. EB planned and carried out all experiments. EB and SS analyzed and interpreted the results, prepared the figures and wrote the paper. AH helped to analyze the windtunnel data. BSH provided intellectual and financial support. All authors critically revised the article.

### Conflict of interest statement

The authors declare that the research was conducted in the absence of any commercial or financial relationships that could be construed as a potential conflict of interest.
